# Extent and pattern of pregnancy losses and progesterone levels during gestation in Swedish Red and Swedish Holstein dairy cows

**DOI:** 10.1186/s13028-018-0420-6

**Published:** 2018-10-30

**Authors:** Sofia Nyman, Hans Gustafsson, Britt Berglund

**Affiliations:** 1 Department of Animal Breeding and Genetics, Centre for Reproductive Biology in Uppsala, Swedish University of Agricultural Sciences, Box 7023, 750 07 Uppsala, Sweden; 2 Department of Clinical Sciences, Centre for Reproductive Biology in Uppsala, Swedish University of Agricultural Sciences, Uppsala, Sweden

**Keywords:** Dairy cow, Embryonic loss, Oestrus, Pregnancy loss, Progesterone

## Abstract

**Background:**

Pregnancy loss is a major source of infertility in dairy cows. Despite a fertilization rate after insemination (AI) of approximately 90%, calving rates are 30%–50%, indicating the occurrence of extensive embryonic and foetal losses. The aim of this study was to establish the extent and pattern of embryonic and foetal loss in Swedish Red (SR) and Swedish Holstein (SH) dairy cows, as well as, the relationship to oestrus intensity (OI) and progesterone (P4) concentration. In total, 2130 AIs and 16,176 milk P4 samples from 359 SR and 212 SH dairy cows were included in the study. Pregnancy losses were estimated using data from P4 values combined with AI information and calving data.

**Results:**

Total pregnancy loss from AI to the day of calving was 65%. Early embryonic loss, late embryonic loss and foetal loss were estimated to be 29, 14 and 13%, respectively. There is strong evidence in the literature that P4 concentrations at different time points are associated with pregnancy loss. In the present study, cows with pregnancy losses had significantly higher P4 levels at the day of AI and significantly lower P4 concentration at days 10, 21 and 30 after AI compared to pregnant cows. Swedish Red cows had significantly lower total pregnancy losses compared to SH cows (62% and 68% respectively, P = 0.017). Early embryonic loss was 6.7% points lower for cows inseminated at a stronger OI (OI = 3) compared to at a weaker OI (OI = 2, P = 0.006). Cows inseminated at ovulation number ≥ 5 had significantly lower early pregnancy losses compared to cows inseminated at first or second ovulation (11.5 and 8% points, respectively, P < 0.05). With an increase of one SD of milk (448 kg ECM) during the first 60 days in milk, early embryonic loss increased by 4.7% points (P = 0.006).

**Conclusions:**

It is important to increase the number of cows calving per insemination by reducing embryo/foetal loss. This outcome can be achieved by management and breeding for optimal P4 levels at critical time points, and by considering oestrus expression in the breeding programmes to facilitate the correct timing of insemination.

## Background

Pregnancy loss is a major cause of infertility in dairy cows and may be the major source of economic loss in the modern dairy system [1]. Despite the high fertilisation success rate in cattle after insemination (AI; ~ 90%), calving rates are significantly lower (30–50%), indicating the occurrence of extensive embryonic and foetal losses during pregnancy [2–4]. The Committee on Bovine Reproductive Nomenclature [5] established the embryonic period from fertilisation to the differentiation stage at approximately 42 days after AI, and the foetal period from day 42 to calving. Moreover, embryonic losses are often categorized as early or late before and after day 25, respectively [4]. Most pregnancy losses occur as embryonic losses. In Holstein–Friesian (HF) dairy cows, early pregnancy losses have been estimated to be 35–45% and late pregnancy losses have been estimated to be less than 10% [4, 6]. In modern high-producing dairy cows more embryos die before day 7 after AI compared to embryos in lower producing cows and heifers [2]. The extent of foetal mortality is not well investigated but is indicated to be in the range of five to 10% [4]. Although the majority of pregnancy losses occur during the early embryonic period, the extent of foetal loss causes higher and more serious economic losses to producers, especially in seasonal production systems, because it is often too late to rebreed the cow [1, 6]. Progesterone (P4) concentrations during both the cycle preceding and following insemination affect embryo survival. There is strong evidence that both excessive and insufficient P4 concentrations at specific time points are negatively associated with pregnancy results [7, 8]. Luteal P4 is essential for preparation of the uterus and oocyte before breeding, as well as for the maintenance of an optimal uterine environment and for supporting the uterus to develop the embryo/foetus during gestation in the cow [9–11]. Changes in P4 during the luteal phase immediately before oestrus and after insemination can cause losses of embryos during days 4–8 after oestrus, during maternal recognition of pregnancy on days 14 through 17 after oestrus, and during the late embryonic period (from day 28 to days 42–50) [12]. Lower P4 concentration 5 days after ovulation may negatively affect priming of the uterus for the arrival of the blastocyst [13] and result in lower pregnancy rates and impaired embryo development [14, 15]. Stronge et al. [16] reported a positive relationship between embryo survival rate and both the P4 measured in milk on day 5–7 after AI and the rate of change in P4 concentrations from day 4–7 after AI. An oestrous cycle, which averages 21 days, is defined as the period initiated by an increase in P4 and subsequently terminated with the next decrease below the threshold value, if the cow is not pregnant. Atypical P4 profiles show deviating P4 concentration pattern, and have been associated with longer calving to first service and calving intervals, as well as reduced conception rates [17–19].

Nyman et al. [19] reported that, cows with a more intensive oestrus have a greater chance to be pregnant at day 60 post AI, and they also reported that Swedish Red cows have stronger oestrous expression and better conception rates compared to SH cows. It remains to be investigated if these findings also result in higher calving rates. Oestrous expression at first ovulation is generally weak or silent, and the conception rates are low [20]. Embryo survival rates, which are assumed to be similar in heifers and low- to moderate-producing dairy cows, but lower in high-producing dairy cows, might not only be an age- or parity-related effect but also a consequence of milk yield level [2]. This is also indicated by Starbuck et al. [9] reporting that heifers maintain more pregnancies than lactating cows and that older cows maintain approximately 9% points less pregnancies than younger cows. However, ageing effects on fertilisation and embryo survival in cows have not been thoroughly investigated.

Many reports have described the incidence and pattern of pregnancy losses in dairy cattle [2], and many studies have assessed a limited number of cows with synchronized oestruses under experimental conditions typically of the HF breed under pastoral conditions or in loose housing systems. However, the aim of this study was to establish the extent and pattern of embryonic and foetal loss after AI in spontaneous oestrus in a long term study of both Swedish Red (SR) and Swedish Holstein (SH) dairy cows, as well as to study the association to circulating P4 levels and P4 patterns, oestrous expression, parity, ovulation number and milk yield.

## Methods

### Animals

Data were collected between January 1992 and December 2008 from a semi-commercial research herd at the Department of Animal Breeding and Genetics, Swedish University of Agricultural Science (Uppsala, Sweden). Altogether, 2130 inseminations with 16,176 milk P4 samples from 359 SR cows and 212 SH cows were included in the study. Milk yield was expressed in kilogram energy corrected milk (ECM) considering fat, protein and lactose contents, using a formula provided by Sjaunja et al. [21]. The average 305 day ECM production increased, between 1992 and 2008, from 8883 kg (SD = 2178 kg) to 10,854 kg (SD = 2657 kg) for SR cows, and from 9585 kg (SD = 2025 kg) to 11,843 kg (SD = 2567 kg) for SH cows. For this study, the milk yield measure used was kg ECM produced during the first 60 days in milk (DIM). Cows were in their 1st to 9th lactation but were grouped 1, 2, and ≥ 3. The distribution of ovulations between the parities were 38.4% (parity 1), 39.7% (parity 2) and 31.9% (parity ≥ 3). The voluntary waiting period was 50 days after calving. From September 1994 to September 2007, the cows were subjected to a calving interval trial during which they were inseminated at the expected calving intervals of either 12 or 15 months. During this trial, 50% of the cows had a pre-planned voluntary waiting period of 140 days. For more information about the calving interval trial, see Ratnayake et al. [22] and Rehn et al. [23].

### Management

Two different indoor housing systems were maintained in the same building at the research herd: a tie-stall system and a loose-housing system with cubicles. All cows were milked twice per day, starting at 06:00 and 15:15 h, in their individual stalls (tie-stall) or in a milking parlour (loose-housing).

Cows were fed according to Swedish standards [24]. From calving until week 16, cows were fed according to the live weight at calving and thereafter on a restricted ration based on production and body condition. Cows were fed roughage and concentrate individually in the tie-stall, whereas only concentrates were fed individually in the loose-housing system. From May to September, all cows were kept on pasture with additional concentrates and roughage fed indoors. From November 2005, a total mixed ratio feeding system was applied.

After calving, cows were visually observed, for oestrous symptoms for approximately 20 min by the experienced research herd staff at three fixed times per day (07:30, 11:30 and 17:00). During May to September, the cows were observed twice daily: during milking in the morning and in the afternoon. Cows were inseminated at the first oestrus after days 50 and 140 for cows with calving intervals of 12 and 15 months, respectively. A maximum of five AIs were allowed per breeding period and the AI period was restricted to a maximum of 130 days. Thereafter, the cow was culled due to infertility. All AIs were performed by experienced AI technicians. Pregnancies were diagnosed within 60 days after the last insemination by rectal palpation in cows not showing any oestrous symptoms and had high milk P4 levels 21 days following the last AI. Reproductive disorders were diagnosed and treated as described in Ratnayake et al. [22]. Culling reasons were recorded and documented.

### P4 measurements

Milk sampling for P4 analysis started during the second week after calving and was sampled twice weekly until cyclicity was detected. Sampling was then reduced to once a week until the first AI and, thereafter at AI, day 10 after each AI and day 21 after each AI until the cows were confirmed pregnant. The following four different kits were used to determine whole milk P4 concentrations; from the start of the collection of data until 1995, the Farmose kit (Orion Diagnostica, Espoo, Finland) was used; between the years 1995 and 1998, the Spectra kit (Orion Diagnostica) was used; from 1998 to the start of December 2007 the Coat-A-Count kit (Diagnostic Products Corporation, Los Angeles, CA) was used; and from December 2007, the Ridgeway kit was used (Ridgeway Science Ltd., Alvington, UK). The intra-assay variation was below 10% for all kits and the corresponding inter-assay variation was below 16% [22, 25]. Progesterone observations from estimated day of ovulation were used to set limits for luteal activity for each of the kits [25]. The P4 concentration that 95% of these estimated days of ovulation fell below was set as the lower limit for luteal activity. The predefined threshold values for the luteal phase for these kits were 25.4, 9.5, 4.1, and 5.0 ng/ml, respectively. The sampling procedure and analyses are further described in Petersson et al. [25].

The P4 concentration at the day of insemination (P4day0), as well as the highest P4 concentration between days 7–13 (P4day10), days 18–24 (P4day21) and days 27–33 (P4day30) were defined and analysed.

Progesterone profiles were generated to describe timely changes of P4. The P4 profiles were classified into a normal profile, which is also a normal oestrous cycle, or the three atypical profiles as follows: (i) delayed cyclicity, (ii) cessation of cyclicity and (iii) prolonged luteal phase. Normal P4 profile was defined as a low P4 value followed by at least two consecutive P4 values above the predefined threshold value, or one P4 value above the predefined P4 threshold and at least 14 days between two consecutive P4 values. Delayed cyclicity was defined as P4 concentrations below the predefined P4 threshold for more than 56 DIM followed by a normal cycle. Prolonged luteal phase was defined as a normal start of cyclicity but with high P4 levels, above the predefined P4 threshold, for at least 20 days, followed by a normal cycle. Cessation of cyclicity was defined as a normal start of cyclicity but interrupted for at least 14 days with P4 concentrations below the predefined P4 threshold value, followed by a normal cycle. More information about the definition of the P4 profiles has been reported by Nyman et al. [26].

### Oestrous expression

The following two oestrous traits were used to describe oestrous expression capacity in normal oestrous cycles: (i) oestrus intensity and (ii) days from calving to first ovulatory oestrus.

Oestrus intensity (OI) was determined using a cumulative score based on an overall scoring system evaluating the oestrus observations, including one or more oestrous symptoms, recorded for each animal by the herd staff. The scoring system was based on the oestrous symptoms and defined using a five point scale where the lower scores were based on local symptoms regarded as more uncertain and the higher scores were based on more classical symptoms, such as stand-to-be-mounted, lowering of the back, and mounting. More information about the scoring systems has been previously been reported by Nyman et al. [20]. In the present study oestrus intensity score one was included in score two (very weak and weak oestrous symptoms) and oestrus intensity score five was included in score four (strong and very strong oestrous symptoms). Score three was defined as normal oestrus intensity. The day of oestrus was defined as the day with the strongest oestrus intensity confirmed by a P4 concentration below the predefined threshold value and a subsequent AI (within 24 h), or as the day with the strongest oestrus intensity confirmed by a P4 concentration below the predefined threshold value (at the same day) and a subsequent commencement of luteal activity (CLA, within 3–8 days). The day of ovulation was defined as 2 days after the day of oestrus confirmed by a P4 concentration below the predefined threshold value 2 days after oestrus, followed by a rise in P4 concentration. Ovulation number was created based on the progesterone concentration.

Days from calving to first ovulatory oestrus (FOO) was defined as the interval from calving to the day of ovulation connected with the first recorded oestrous symptoms.

Distribution of percentage and number of inseminations, days from calving to first ovulatory oestrus, oestrus intensity (2 = weak and 4 = strong), oestrus detection rate and number of false inseminations (at high P4 concentration) per ovulation number are shown in Table 1.

**Table 1:** Distribution of number and proportions of inseminations, days from calving to first observed ovulatory oestrus, oestrus intensity (2 = weak and 4 = strong), number and proportions of detected oestruses and number and proportions of false inseminations per ovulation number for Swedish dairy cows

	Ovulation number				
	1	2	3	4	≥ 5
Inseminations, n (%)	70 (3.3)	370 (17.4)	524 (24.6)	439 (20.6)	727 (34.1)
Day of first observed ovulatory oestrus, mean (SD)	46 (29)	67 (33)	92 (34)	117 (34)	166 (45)
Oestrus intensity, mean (SD)	2.21 (1.34)	2.91 (0.73)	2.93 (0.67)	2.94 (0.68)	2.97 (0.68)
Detected oestruses, n (%)^a^	657 (75)	614 (77)	686 (83)	541 (87)	911 (89)
False inseminations, n (%)^b^	9 (11.4)	17 (4.4)	21 (3.9)	12 (2.7)	25 (3.3)

^a^Number and proportion of visible oestrous observations out of oestruses based on low P4 concentration

^b^Inseminations performed with high P4 concentration (numbers and proportions out of total number of inseminations)

### Pregnancy losses

Pregnancy losses were estimated using data from P4 values combined with information from 2130 inseminations from which four traits were defined as follows: (i) early embryonic loss, (ii) late embryonic loss, (iii) foetal loss, and (iv) total pregnancy loss. Approximately 3.7% (n = 84) of the inseminations was excluded as a cause of false inseminations, i.e. inseminations performed in the presence of high P4 concentration at the day of AI.

Early embryo loss was defined as the proportion of cows that lost their pregnancy between days 1 and 24 after AI, based on the number of cows with low P4 concentrations or repeated inseminations up to day 24, divided by the total number of inseminated cows at AI. At day 25, a total of 619 inseminations were defined as early embryonic loss.

Late embryo loss was defined as the proportion of cows that lost their pregnancy between days 25 and 60 after AI, based on the number of cows with low P4 concentrations or repeated inseminations from day 25 up to day 60, divided by the total number of cows that remained pregnant at day 25. Six cows were culled due to fertility failure between days 25 and 60 after AI and were considered as late embryonic loss. At day 61, a total of 273 inseminations were defined as late embryonic loss.

Foetal loss was defined as the proportion of cows that lost their pregnancy from days 61 after AI to calving, based on the number of cows with low P4 concentrations or repeated inseminations from day 61 to calving, divided by the total number of cows that remained pregnant at day 61. Eleven and 102 cows were culled due to abortion and fertility failure, respectively, between days 61 after AI and calving, and they were considered as foetal loss. At the day of calving, 171 inseminations were resulting in foetal losses.

Total pregnancy loss was defined as the proportion of cows that lost their pregnancy from day 1 after AI to calving, divided by the total number of cows that remained pregnant until calving. During the observed periods for pregnancy losses, 341 cows were excluded because of culling reasons not related to fertility. The inseminations from these culled cows were excluded immediately after the period in which the cows were culled and not included in the total pregnancy loss. At the subsequent day of calving, 1789 inseminations remained in the dataset, and 1063 of these were defined as total pregnancy loss.

### Statistical analysis

The data were analysed using mixed linear models via the MIXED procedure in SAS 9.4 [27]. The statistical model included the fixed effects of breed (SR and SH), parity (1, 2 and ≥ 3), calving trial (12 and 15 months), ovulation number (1, 2, 3, 4 and ≥ 5), housing system (tie-stall and loose-housing), insemination year (1992–2008) and insemination season (January to April; May to September; and October to December). Milk yield was considered as a fixed regression and cow nested within breed was considered as a random effect. The results are presented as least square mean ± SE, and the t-test was used when comparing the sub-classes of main effects. In all analyses, differences were considered significant when P ≤ 0.05, whereas differences between P > 0.05 and P ≤ 0.10 were considered tendencies. The categorical traits OI and the atypical P4 profiles were also analysed using GLIMMIX procedure in SAS [27]. Because there were no differences in the results between the two methods, however, the results from the MIXED procedure were presented.

When delayed cyclicity or cessation of cyclicity was included as independent variables in the model, information from the ongoing P4 profiles was used. When prolonged luteal phase was included as an independent variable in the model, information from the previous P4 profiles was used.

## Results

### Pregnancy losses

Total pregnancy loss from AI to the day of calving was 65.1%. Early embryonic loss, late embryonic loss and foetal loss were estimated to be 29.0, 14.3, and 13.0%, respectively. Out of the 619 AIs performed during the period of early pregnancy loss days 1–24 after AI, the P4 concentration was low at day 10 after AI in 171 oestrous cycles indicating a very early loss of 7.4% as a part of the early embryonic loss.

Early embryonic loss was 6.7% points lower when AIs were performed at normal oestrus intensity (OI = 3) compared to at a weak oestrus intensity (OI = 2, P > 0.001). Pregnancy loss was not significantly associated with FOO.

Compared to SH cows, SR cows tended to have lower late embryonic loss and foetal loss (Table 2), and they had 5.5% points lower total pregnancy loss (P > 0.02).

**Table 2:** Least square means (SE) for early embryonic loss, late embryonic loss, foetal loss and total pregnancy loss for Swedish Red (SR) and Swedish Holstein (SH) cows

Effect	Total n_obs_	Early embryonic loss (%)	Late embryonic loss (%)	Foetal loss (%)	Total pregnancy loss (%)
Breed					
SR	1343	29.1 (2.72)	13.5 (1.98)	12.4 (1.66)	62.4 (2.85)^a^
SH	786	28.8 (2.92)	15.2 (2.10)	13.7 (1.76)	67.9 (3.02)^b^
Parity					
1	893	30.5 (3.04)	14.5 (2.04)	11.8 (1.68)^a^	58.7 (2.94)^a^
2	620	28.0 (3.119)	13.7 (2.22)	11.9 (1.82)^a^	64.1 (3.20)^b^
≥ 3	616	28.4 (3.21)	14.9 (2.22)	15.4 (1.84)^b^	72.6 (3.18)^c^
Calving interval (months)					
12	1390	30.8 (2.56)	13.4 (1.86)	14.9 (1.56)^a^	66.3 (2.68)
15	739	27.2 (3.12)	15.2 (2.27)	11.2 (1.90)^b^	64.0 (3.26)
Ovulation number					
1	70	34.8 (6.16)^ab^	12.3 (4.40)	16.2 (3.53)^ab^	67.7 (6.36)
2	370	32.1 (3.30)^a^	15.7 (2.42)	10.5 (1.98)^a^	67.1 (3.50)
3	524	27.7 (2.98)^ab^	15.7 (2.19)	11.0 (1.80)^a^	64.3 (3.15)
4	439	26.9 (3.03)^ab^	14.5 (2.23)	12.9 (1.82)^ab^	63.1 (3.20)
≥ 5	726	23.3 (2.62)^b^	13.6 (1.91)	14.6 (1.59)^b^	63.6 (2.74)
Housing					
Tie-stall	911	30.5 (2.15)	12.6 (1.50)	9.4 (1.26)	62.1 (2.16)
Loose-housed	1130	28.5 (1.95)	13.0 (1.38)	8.0 (1.16)	60.3 (1.99)
Insemination season					
January–April	1022	30.5 (2.68)^a^	14.0 (1.95)	10.3 (1.61)^a^	64.6 (2.81)
May–September	532	25.0 (3.12)^b^	14.6 (2.24)	17.4 (1.85)^b^	68.4 (3.23)
October–December	575	31.3 (3.28)^a^	14.5 (2.38)	11.4 (1.97)^a^	62.5 (3.42)

^a–c^Means within effect and column with different superscript are significantly different P ≤ 0.05

Foetal loss and total pregnancy loss increased with increasing parity (Table 2). Cows in parity ≥ 3 had 3.4% points higher foetal loss (P > 0.02) and 8.5% points higher total pregnancy loss (P > 0.01) compared to cows in parity two and total pregnancy loss was 5.4% points higher for cows in parity two compared to cows in parity one (P = 0.04). Total pregnancy loss was 13.9% points higher in multiparous cows (parity ≥ 3) than in primiparous cows (Table 2).

Cows with a planned calving interval of 12 months (voluntary waiting period = 50 days) had significantly higher foetal loss (3.7% points) compared to cows with a 15 months calving interval (voluntary waiting period = 140 days, Table 2).

**Fig. 1:**
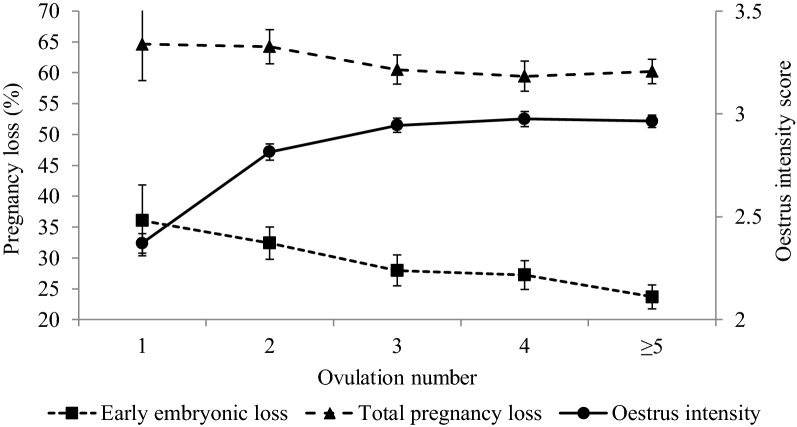
Least square means (SE) for oestrus intensity (solid line; right hand y-axis, 2 = weak to 4 = strong), early embryonic loss in (dotted line and square markers; left hand y-axis) and total pregnancy loss (dotted line and triangle markers; left hand y-axis) by ovulation number for Swedish Red and Swedish Holstein cows

Early embryonic loss and total pregnancy loss had a tendency to decrease with increasing cycle number (Fig. 1), from 34.8% early embryonic loss and 67.7% total pregnancy loss in cycle number one to 23.3% early embryonic loss and 63.6% total pregnancy loss in cycle number five and higher (Table 2; Fig. 1). Cows inseminated at ovulation number five and higher had significantly lower early embryonic loss compared to cows inseminated at first and second ovulation (11.5 and 8.8% points respectively; Table 2).

Cows inseminated at pasture (May to September) had significantly lower early embryonic loss (5.5 and 6.3% points, respectively) and significantly higher foetal loss (7.1 and 6.0% points respectively), compared to cows inseminated in January to April or October to December (Table 2).

Early embryonic losses were significantly associated with milk yield. With an increase of one SD of milk (447.5 kg ECM) during the first 60 DIM, early embryonic loss increased with 4.7% points (P > 0.01).

Housing system, atypical P4 profile and insemination year (not presented) had no significant effects on any of the pregnancy losses.

### Progesterone concentrations at day of insemination, and days 10, 21 and 30 after AI

Progesterone levels for pregnant cows and cows with pregnancy losses at the day of AI (P4day0), day 10 (P4day10), day 21 (P4day21) and day 30 (P4day30) after AI are summarized in Table 3. In general, cows with pregnancy losses had significantly higher P4 levels at the day of AI compared to pregnant cows. Cows with pregnancy loss between days 1 after AI and calving had significantly lower P4 concentrations at days 10, 21 and 30 after AI compared to subsequent calving cows.

**Table 3:** Least square means (SE) for progesterone concentrations at the day of insemination (P4day0), day 10 (P4day10), day 21 (P4day21) and day 30 (P4day30) after AI for pregnant cows and cows subjected to pregnancy losses (non-pregnant) at days 1–24, 25–60 and 61-calving after AI for Swedish dairy cows

Pregnancy loss at day	Progesterone concentration (ng/ml) (SE)							
	P4day0		P4day10		P4day21		P4 day 30	
	Non-pregnant	Pregnant	Non-pregnant	Pregnant	Non-pregnant	Pregnant	Non-pregnant	Pregnant
1–24	2.37 (0.20)	2.01 (0.16)	16.90 (0.42)^a^	19.97 (0.31)^b^	10.43 (0.68)^a^	27.90 (0.39)^b^	–	–
25–60	3.14 (0.28)^a^	2.10 (0.19)^b^	18.11 (0.57)^a^	20.46 (0.32)^b^	22.56 (0.78)^a^	28.84 (0.42)^b^	12.89 (1.37)^a^	32.08 (1.03)^b^
61-calving	2.56 (0.27)^a^	1.42 (0.16)^b^	21.18 (0.76)	20.35 (0.36)	25.94 (1.08)^a^	29.22 (0.43)^b^	27.74 (1.98)^a^	32.22 (1.10)^b^
1-calving	1.70 (0.16)^a^	1.32 (0.19)^b^	17.66 (0.39)^a^	19.64 (0.47)^b^	18.86 (0.60)^a^	28.69 (0.67)^b^	20.30 (0.86)^a^	30.72 (0.97)^b^

^a, b^Means within interval (days 1–24 after AI, days 25–60 after AI, day 61 after AI to calving, and 1 day after AI to calving) with different superscript are significantly different P ≤ 0.05

**Fig. 2:**
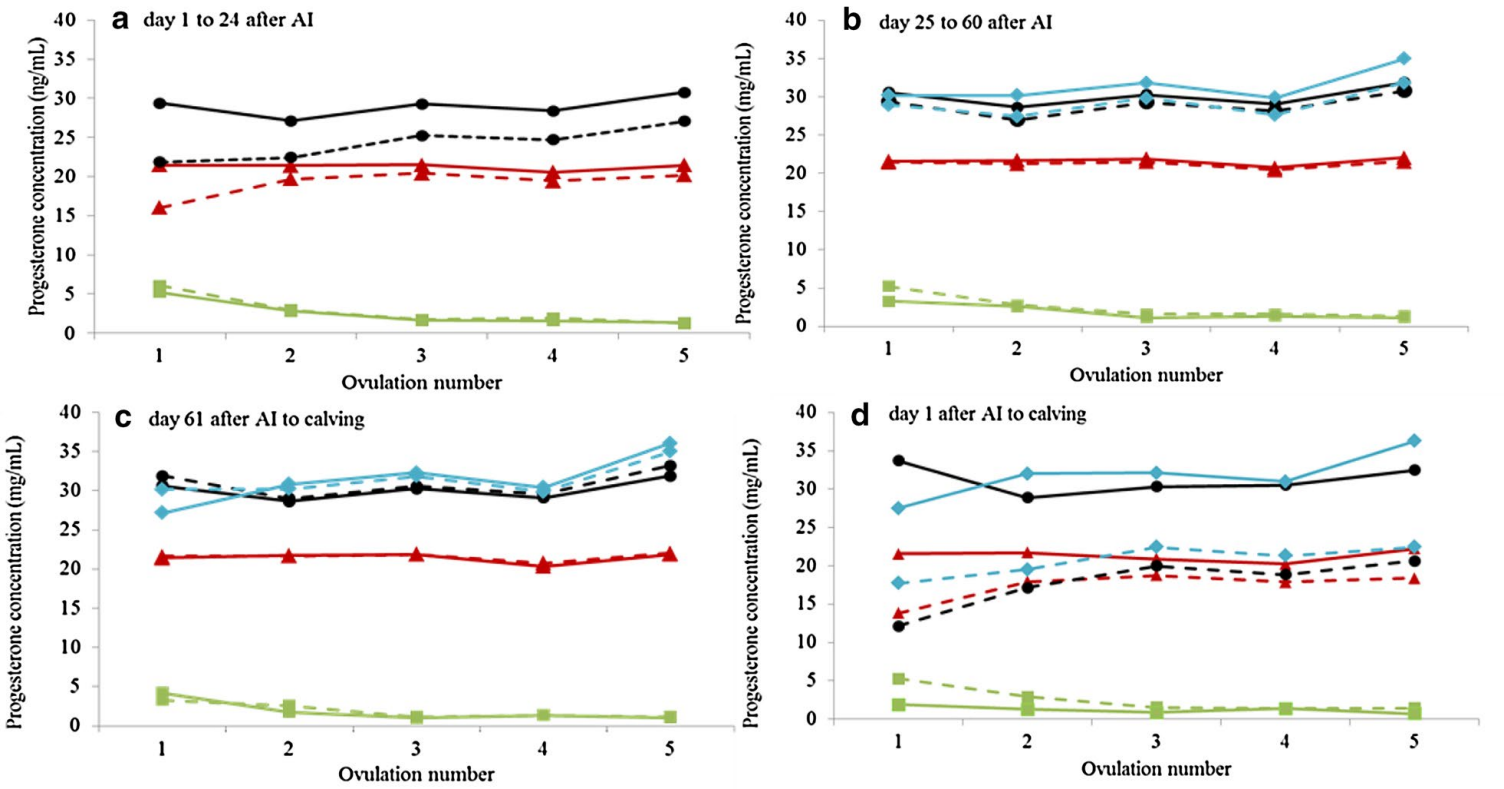
Progesterone concentration at insemination (green line), at day 10 (red line), at day 21 (black line), and at day 30 (blue line) after AI by ovulation number for cows with pregnancy losses (dotted line) and pregnant cows (solid lines) during the following periods: **a** days 1–24 after AI, **b** day 25–60 after AI, **c** day 61 after AI to calving, and **d** day 1 after AI to calving

Figure 2a–d illustrates the P4 levels at the day of AI and days 10, 21 and 30 after AI by increasing ovulation number (1 to ≥ 5) for pregnant cows and cows with pregnancy losses in the following periods: (a) 1–24 days after AI, (b) 25–60 days after AI, (c) day 61 after AI to calving, and (d) day one after AI to calving. At the time of insemination, the P4 concentration was significantly higher in cycle numbers one and two compared to the other cycles for both pregnant cows and cows with pregnancy losses. With increasing ovulation number (Fig. 2a–d), P4 levels at AI decreased and P4 levels at days 10, 21 and 30 after AI increased, for both pregnant cows and cows with pregnancy losses for all periods.

## Discussion

In this study we estimated the reproductive losses after insemination in spontaneous oestruses. In contrast, several earlier studies have reported pregnancy results in synchronized oestruses of the HF dairy breed [4, 28]. To our knowledge the extent and timing of reproductive losses in the two main dairy breeds, SR and SH, have not yet been investigated. The present dataset include records of oestruses, inseminations, P4 levels, pregnancy checks, abortions and calving information under field conditions in a semi-commercial dairy herd.

Our results showed that the expected outcome from 100 inseminations performed in dairy cows in spontaneous true oestrus (low P4 levels) is a calving rate of approximately 35–40%. In general, our results are comparable with data reported from British- and Holstein–Friesian cows managed under pastoral conditions in Ireland [2], but higher than the 28% calving rate in high-producing dairy HF cows in the USA estimated by Santos et al. [4]. In the present study, early embryonic loss was estimated to be 29% accounting for the majority of the pregnancy losses. Late embryonic loss was estimated to be 14%, and foetal loss was estimated to be 13%, which is in accordance with previous studies in high-producing dairy cows in temperate climate. Humblot [29] evaluated embryonic losses in Holstein cows in 44 herds in France by determine cows as pregnant if P4 < 3.5 ng/ml at day 0 (at AI), P4 > 5 ng/mL at day 21 and 24 after AI, Pregnancy Specific Protein measurements detectable, absence of a second insemination, and/or pregnant at rectal palpation. They observed that early embryonic loss after first AI was 31.6% and that late embryonic loss was 14.7%. Late embryonic mortality after day 27 of gestation has been found to range from 3.2% in dairy cows producing 6000–8000 kg of milk per year in Ireland [30] to up to 42.7% in high-producing cows under heat stress [31]. In cattle, foetal losses (> 42 days) are investigated less than early and late embryonic losses. Sub-luteal P4 levels 10 days after AI was found in 7.4% of the cycles, which indicated a very early reproductive loss. There may be two possible explanations for a low P4 level at day 10 after AI, these are: ovulation and subsequent fertilisation failure or early regression of the corpus luteum despite a successful fertilisation or not. These possibilities together with the fact that a normal P4 profile at day 10 post AI do not exclude fertilisation failure and do not allow calculation of an overall fertilisation failure as a part of the early reproductive loss. In low- and moderate-yielding dairy cows, fertilisation failure rates are in the order of less than 10% following the use of high-quality semen, but based on published results, the failure rates are higher in high-producing dairy cows [2].

Early embryonic loss decreased with stronger oestrous expression, which was not surprising because our earlier study showed a positive association between oestrus intensity and conception rates [19]. These results are also in agreement with Pereira et al. [28], who reported that a decrease in pregnancy losses (between 32 and 60 days of pregnancy) associates with cows expressing oestrus compared to no oestrus using protocols for timed insemination or timed embryo transfer. A more intensive oestrus will most likely improve the timing of AI in a spontaneous oestrus. Dransfield et al. [32] showed that the highest conception rates for AI occur between four and 12 h after the onset of standing oestrus or 12–24 h before ovulation. Moreover, Saacke et al. [33] showed that early inseminations results in low fertilisation rates but good embryo quality, whereas late inseminations result in high fertilisation rates but poor embryo quality. Our results showed a positive effect of oestrus intensity, and possibly AI timing, on embryo survival before day 25 after AI. This may indicate that the timing of fertilisation and/or interferon-tau signalling by the embryo on day 17 are important biological mechanisms [34].

The lower total pregnancy loss for SR cows is in agreement with our earlier study where we found higher conception rates in SR cows than in SH cows based on transrectal pregnancy checks at approximately day 60 after AI [19]. Data on the effect of breed are very sparse in the literature. Diskin et al. [35] found a rate of early embryonic death in HF cows of 43% in 2006 compared to 28% in British-Friesian cows in 1980. This difference may suggest a breed effect but may also indicate a deterioration of the embryo survival rate by year.

Our results showed a higher pregnancy loss by parity, which is in agreement with earlier results [9, 36], suggesting that the age of the mother may increase the risk of pregnancy losses. While fertility in general is regarded higher in heifers than in cows, there is not much information about age effects in cows. Balendran et al. [37], however, studied 163 Canadian Holstein cows and heifers and reported a clear parity effect on pregnancy rates at first insemination of, 68, 43, 20 and 12% in heifers and in cows in parity 1, 2 and 3–4, respectively. It is possible that ageing effects of the uterus can exist depending on true physiological effects or as a result of acquired dysfunctions such as subclinical infections [38].

We did not find a general association between FOO and pregnancy loss in our study. However, cows with a voluntarily waiting period of 50 days compared to 140 days had higher foetal loss. This is in line with studies by Ball and Peters [39] who observed lower conception rates in cows inseminated before 50 DIM, and by Humblot [29] who reported lower late embryonic loss (13%) for cows inseminated after day 70 postpartum compared to cows inseminated before 70 days after calving (17%). This indicates that longer planned calving intervals may be beneficial for embryo and foetal survival but suboptimal for other reasons.

Our results showed a decrease in pregnancy loss by ovulation number together with increased oestrous expression and higher luteal P4 levels. A repeated P4 priming of the uterus by cycle induces higher oestradiol secretion by the pre-ovulatory follicle, which creates an optimal environment for follicle maturation and prepares the uterus for embryo support [10]. Higher P4 levels stimulate the growth of the embryo during the critical period of maternal recognition of pregnancy [9–11].

The extent of early embryonic loss was associated with a higher milk yield during the first 60 DIM. This result is in agreement with a review by Diskin et al. [2] reporting a greater embryonic loss for high-producing dairy cows compared to low-yielding cows and heifers. However, this result disagrees with the review of several investigations by Santos et al. [4] stating that there is little or no indication that milk production is a risk factor for increased pregnancy losses in dairy cattle. It is difficult to compare the results of our study with the results of previous studies, due to the great differences in both the study design and in the definition of milk production. Increased milk yield is generally associated with increased feed intake and metabolism of P4 in the liver [36, 40]. A low amount of circulating P4 may lead to a slower rise in P4 during early dioestrus, which may reduce the development of the early embryo and increase the risk of pregnancy losses in high-producing dairy cows. Nevertheless, in our study no association between milk yield and late embryonic loss and foetal loss was observed, which agrees with findings by others [36, 41].

Cows with pregnancy losses had higher P4 concentration at the day of AI compared to pregnant cows which is in accordance to a study by Bruinjé et al. [42] studying 115 primiparous and 249 multiparous Canadian Holstein cows. Suprabasal P4 concentrations at AI have been found to impair fertility [43] and have led to repeated AIs and prolonged calving intervals [44]. High P4 concentration around AI may lead to reduced fertilisation rates, blocked ovulation or may affect the sperm transport [45]. We also observed that P4 concentrations at AI in the first and second oestrous cycles were higher compared to in later cycles. Insemination of cows at later ovulations, for which also a stronger oestrous expression was found, may increase the chance of successful pregnancies.

Cows with pregnancy losses had lower P4 concentration during gestation compared to pregnant cows, which is in accordance to earlier studies [9, 11, 46]. However, in contrast to this a recent study by Bruinjé et al. [42] reported that cows with pregnancy losses had higher P4 values compared to pregnant cows. The requirement of P4 after AI for inducing a uterine environment supporting embryo growth, implantation, and maintenance of pregnancy is unequivocal. However, there are conflicting data in the literature on relationships between certain levels of P4 and fertility. In recent studies relationships between P4 levels early after AI and embryo survival have been reported [46]. Hommeida et al. [47] studied 19 lactating HF cows in Japan and reported higher P4 concentration in pregnant cows after day 10 compared to cows returning to oestrus before day 25.

## Conclusions

Due to reproductive losses throughout the gestation period, only one-third of cows that are inseminated actually calve. One general physiological reason could be suprabasal P4 levels to support fertilisation or inadequate systemic levels of progesterone to support embryo/foetal survival. We found associations between pregnancy losses and several animal-environment management factors, e.g., ovulation number and planned calving interval. To delay the start of insemination to later ovulations may not always be applicable and economically relevant because it may delay the first insemination and result in longer calving intervals.

The major challenge for future research will be to increase pregnancies per AI by both genetic and management improvements. According to our results, more focus on oestrous expression traits would benefit early embryo survival. A more precise knowledge about the timing and possible background could improve management for increased embryo survival. Combined with more powerful breeding tools, such as genomic selection, this may further reduce embryonic losses.
